# Effect of an interactive cardiopulmonary resuscitation assist device with an automated external defibrillator synchronised with a ventilator on the CPR performance of emergency medical service staff: a randomised simulation study

**DOI:** 10.1186/s13049-017-0379-8

**Published:** 2017-04-04

**Authors:** Rainer Nitzschke, Christoph Doehn, Jan F. Kersten, Julian Blanz, Tobias J. Kalwa, Norman A. Scotti, Jens C. Kubitz

**Affiliations:** 1 Department of Anesthesiology, Center of Anesthesiology and Intensive Care Medicine, University Medical Center Hamburg-Eppendorf, Martinistrasse 52, 20246 Hamburg, Germany; 2 Department of Medical Biometry and Epidemiology of the University Medical Center Hamburg-Eppendorf, Hamburg, Germany; 3 WEINMANN Emergency Medical Technology, Hamburg, Germany

**Keywords:** Cardiopulmonary resuscitation, Advanced life support, Emergency medical service, Education and simulation, CPR assist devices

## Abstract

**Background:**

The present study evaluates whether the quality of advanced cardiac life support (ALS) is improved with an interactive prototype assist device. This device consists of an automated external defibrillator linked to a ventilator and provides synchronised visual and acoustic instructions for guidance through the ALS algorithm and assistance for face-mask ventilations.

**Methods:**

We compared the cardiopulmonary resuscitation (CPR) quality of emergency medical system (EMS) staff members using the study device or standard equipment in a mannequin simulation study with a prospective, controlled, randomised cross-over study design. Main outcome was the effect of the study device compared to the standard equipment and the effect of the number of prior ALS trainings of the EMS staff on the CPR quality. Data were analysed using analyses of covariance (ANCOVA) and binary logistic regression, accounting for the study design.

**Results:**

In 106 simulations of 56 two-person rescuer teams, the mean hands-off time was 24.5% with study equipment and 23.5% with standard equipment (Difference 1.0% (95% CI: −0.4 to 2.5%); *p* = 0.156). With both types of equipment, the hands-off time decreased with an increasing cumulative number of previous CPR trainings (*p* = 0.042). The study equipment reduced the mean time until administration of adrenaline (epinephrine) by 23 s (*p* = 0.003) and that of amiodarone by 17 s (*p* = 0.016). It also increased the mean number of changes in the person doing chest compressions (0.6 per simulation; *p* < 0.001) and decreased the mean number of chest compressions (2.8 per minute; *p* = 0.022) and the mean number of ventilations (1.8 per minute; *p* < 0.001). The chance of administering amiodarone at the appropriate time was higher, with an odds ratio of 4.15, with the use of the study equipment CPR.com compared to the standard equipment (*p* = 0.004). With an increasing number of prior CPR trainings, the time intervals in the ALS algorithm until the defibrillations decreased with standard equipment but increased with the study device.

**Conclusions:**

EMS staff with limited training in CPR profit from guidance through the ALS algorithm by the study device. However, the study device somehow reduced the ALS quality of well-trained rescuers and thus can only be recommended for ALS provider with limited experience.

## Background

The quality of out-of-hospital cardiopulmonary resuscitation (CPR) is an important link in the chain of survival and has a direct impact on patient outcomes after out-of-hospital cardiac arrest (OHCA) [1–4]. Nevertheless, both survival to hospital discharge and acceptable neurologic outcome after cardiac arrest remain poor [5, 6]. Although the outstanding importance of the quality of CPR after OHCA has been known for 20 years, the quality of both out-of-hospital and in-hospital resuscitations is often far behind the recommendations of resuscitation guidelines because both emergency medical service (EMS) staff members and in-hospital emergency teams have difficulty with complying with these guidelines [4, 7–12].

According to the 2015 CPR guidelines of the European Resuscitation Council (ERC), rescuers should deliver chest compressions (CC) at a rate of 100–120 compressions per minute, analyse the electrocardiographic (ECG) rhythm as soon as possible, try to change the person doing chest compressions every 2 min, apply ventilations with a compression to ventilation ratio of 30:2 and administer intravenous medication according to cardiac rhythm. Additionally, rescuers should minimise the hands-off time to avoid interrupting CC and should not interrupt CC for more than 10 s. to provide ventilation [3, 13]. Resuscitation guidelines emphasise the importance of limiting the hands-off time to a minimal duration [3, 13].

The hands-off time is due to pausing CC when checking for vital signs, conducting cardiac rhythm analysis, applying bag-mask ventilations, establishing venous access for emergency medication and providing advanced airway management. EMS staff require regular training on the advanced life support (ALS) algorithm, according to resuscitation guidelines [3, 14]. But then, exposure to OHCA is rare, especially in rural areas, and some teams thus have limited experience in ALS [15]. There are various devices commercially available that have been developed to increase the quality of CPR, including metronome, visible and audible feedback systems and active chest compression-decompression assist devices.

The goal of the present study was to evaluate whether the quality of ALS performed by experienced EMS staff could be improved with process assistance from a new interactive prototype device that consists of an automated external defibrillator (AED) linked to a ventilator and provides synchronised visual and acoustic instructions for ALS workflow measures and assistance for face-mask ventilations. We determined the effect of the prototype assist device compared to the standard equipment and the effect of the number of prior ALS trainings of the EMS staff on the CPR quality in a simulation study. The following null hypothesis was tested: the prototype assist device and the number of prior ALS trainings do not affect the CPR performance of the EMS staff in the simulations.

## Methods

### Study design and participants

We compared the standard CPR equipment (standard equipment) to the interactive device (CPR.com device) in a full-scale mannequin simulation study with a prospective, controlled, randomised cross-over study design. The regional ethics committee of the Medical Council waived the requirements for ethical approval on January 28, 2014.

Data were collected during two standardised simulated cardiac arrest scenarios in three different rescue stations in the Hamburg urban area. Participants were experienced, practicing EMS staff members but had three different levels of vocational education: paramedics, emergency medical technicians (EMTs) and EMTs who were in vocational training to become paramedics. All participants were employees of the G.A.R.D. Ambulance Service in Hamburg, Germany. Recruitment of study participants was on a voluntary basis. There was no selection of volunteering participants. All participants gave written informed consent to participate in the study and evaluate their CPR performance data. All volunteers were regularly trained in ALS for re-certification.

### Study protocol

Prior to the study measurements, participants were made familiar with the study protocol, the standard equipment and the CPR.com study device. This education included a short recapitulation of the ALS algorithm of the ERC CPR guidelines, an introduction of the study equipment and instructions on the use of the devices in the study scenario. Participants were instructed to use a bag-valve-mask device or the CPR.com device, to insert a laryngeal tube (LT) in the course of the CPR scenario and to use a mechanical ventilator (standard ventilator or CPR.com device) after securing the airway with a LT.

Each team of two rescuers used the standard equipment in one of the two simulation scenarios and the study equipment in the other. Block randomisation was employed to assign the sequence in which the two treatment scenarios were applied to each rescuer team. A block consisted of four rescuer teams. Immediately prior to the simulation, each team received a sealed envelope containing information on the sequence to be employed.

In all simulation scenarios, participants had to perform ALS on a high-fidelity, full-scale training mannequin (Resusci Anne Simulator, Laerdal Medical AS, Stavanger, Norway) that was placed on the floor, which simulated a patient with OHCA due to persisting ventricular fibrillation (VF).

Each simulation scenario was terminated after the fourth defibrillation shock was administered. The participants had a rest of at least 20 min between the CPR sessions in the study.

### Equipment

In all CPR scenarios, the rescuer teams had to use basic equipment, which consisted of tools for establishing peripheral venous access, infusion sets, infusion fluids, syringes, glass ampoules with epinephrine and amiodarone, cannulas, stethoscope, a LT (LTS-D Size 4, VBM Medizintechnik GmbH, Sulz, Germany) for airway management and a portable suction unit. Additionally, participants were randomised to use either the standard or study equipment in each of their CPR scenarios.

### Standard equipment

The standard equipment consisted of an AED MEDUCORE Standard (WEINMANN Emergency Medical Technology, Hamburg, Germany) and a ventilator MEDUMAT Standard^2^ (WEINMANN Emergency Medical Technology) fixed together with a 2-l oxygen bottle on a portable system. The MEDUCORE Standard provided an acoustic metronome of 100/min and announcements prompting the start of CC and rhythm analysis to assist BLS measures.

In the standard equipment CPR session, the MEDUMAT Standard^2^ was used for ventilation via the inserted LT. At the start of the ventilator in case of a CPR, the participants had to select the type of patient to be ventilated from a menu, which presented the options of an adult patient, an older child or a toddler. After selecting an adult patient, the ventilator applied volume-controlled intermittent positive pressure ventilation (IPPV) with a pre-set frequency of 12/min, a tidal volume (Vt) of 500 ml, a maximum inspiratory pressure (Pmax) of 30 mbar without positive end expiratory pressure (PEEP) and a ratio of inspiration to expiration (I:E) of 1:1.7. Before inserting the LT, the teams provided face-mask ventilations with a bag-valve-mask device (Combibag™, WEINMANN Emergency Medical Technology).

### Study equipment (CPR.com)

**Fig. 1:**
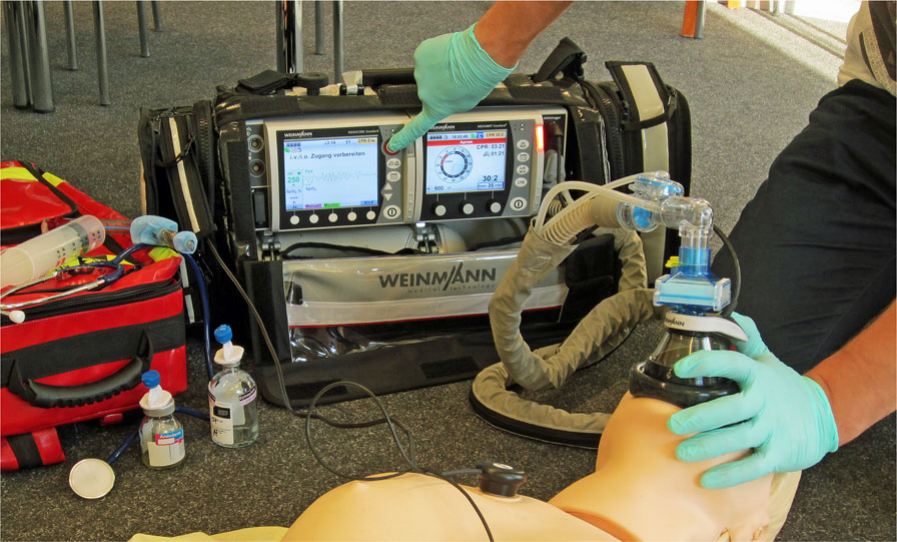
The prototype device CPR.com (WEINMANN Emergency Medical Technology; Hamburg, Germany) with the switch for triggering ventilations via the face mask (MEDUtrigger™, WEINMANN Emergency Medical Technology) in the foreground and a rescuer communicating with the device

The CPR.com equipment (WEINMANN Emergency Medical Technology) was a prototype device that was based on the technology of the AED MEDUCORE Standard and the ventilator MEDUMAT Standard^2^ fixed on a portable system that were linked to and synchronised with each other. CPR.com provided a metronome of 100/min and instructions on BLS and ALS measures, according to the CPR guidelines, both visually on the screen and by voice announcements. These instructions prompted the rescuers to perform ALS measures according to the ERC guidelines and thereby guided BLS, AED use, changeover of the rescuers’ roles and places, airway management, venous access, and the preparation and administration of adrenaline (epinephrine) and amiodarone, if appropriate. After the requested ALS measure was performed, the rescuers confirmed the completion of the ALS measure on the device’s user interface (Fig. 1).

The CPR.com device provided ventilation via a face-mask and a secured airway. Similar to the standard equipment, the rescuers selected the type of patient from a menu. If the participants selected an adult patient, the CPR.com device was automatically adapted to the settings of Vt 500 ml, Pmax 30 mbar, PEEP 0 mbar and I:E 1:1 for face-mask ventilations. These inflations were triggered by a switch at the face-mask (MEDUtrigger), which was connected to the device by a ventilation tube. CPR.com warned the provider in case that the MEDUtrigger had not been used for more than 40 s. After the placement of the LT was confirmed on the device, CPR.com automatically started IPPV with a pre-set frequency of 12/min, a Vt of 500 ml, Pmax 30 mbar, PEEP 0 mbar and I:E of 1:1.7.

The CPR.com device paused ventilations automatically during ECG rhythm analysis and defibrillations.

The rescuers were not familiar with the standard and the study equipment prior to the study.

### Data collection

The simulation scenarios were digitally video recorded and analysed for the BLS and ALS measures taken by the participants. The ventilation data were recorded using a differential pressure and flow sensor (Hamilton Medical, Bonaduz, Switzerland) placed between the mannequin’s upper airway and lungs. After completing the two simulations, the study participants were asked to complete a questionnaire on their demographic data, vocational education, CPR experience, prior training in CPR and handling of the CPR.com device.

### Outcome measures

The quality of CPR and the degree of CPR guideline compliance of the rescuer teams were measured using prospectively defined primary and secondary outcome variables. The primary endpoint was the hands-off time, which is defined as the percentage of the entire CPR time without CC. Secondary endpoints were the frequency of CC and the total number of CC and ventilations per minute, the number of changes of the person doing CC (rescuer’s change in compressor and ventilator roles) and the proportions of CPRs with adrenaline and amiodarone applied to the correct point in time according to ERC guidelines. Depth and release of CC could not be evaluated, as they are not accurately registered by the software version used in the mannequin when CC and ventilations are applied simultaneously.

Additionally, secondary endpoints were the time intervals until the first, second and third defibrillations, intubation with the LT, implementation of venous access and first administration of adrenaline and amiodarone.

### Statistical analysis

The categorical data are presented as counts (percentages) and the continuous data as means with standard deviation (SD), standard error (SE) or 95% confidence interval (CI). We used hierarchical multivariate analyses of covariance (ANCOVA) adapted for a cross-over study design. Binary logistic regression models were used to determine the effect of the study device (CPR.com) on the administration of adrenaline and amiodarone at the correct point in time. All analyses were done using mixed model approaches, reflecting the fact that we have firmly established two-person rescuer teams conducting the same type of investigation two times, each time under a different treatment regime. The rescuer team was therefore considered as random effect. Treatment (equipment), period (to determine carry-over effects) and sequence (i. e. which treatment was administered first) were fixed effects in the models. Cumulative number of prior CPR training sessions attended by either of the team members within the last 5 years were incorporated as covariates in the analyses as well. Additionally, we included the highest vocational education level of the two partners in a rescuer team as a covariate in the model. Sample size calculation was targeted to detect a minimal relevant difference of 5% in the hands-off time, with a power of 80% and a significance level of 5%. The assumed standard deviation of 7% was based on the results of similar studies, and a need for 98 evaluable simulations was found [16, 17]. Based on this number of simulations and accounting for a 12% drop-out rate, the required number of simulations was set at 112.

*P*-values below 0.05 were considered significant, without correcting for multiple testing. Statistical analysis was performed using the statistical software package R 3.2.3 [18].

## Results

**Fig. 2:**
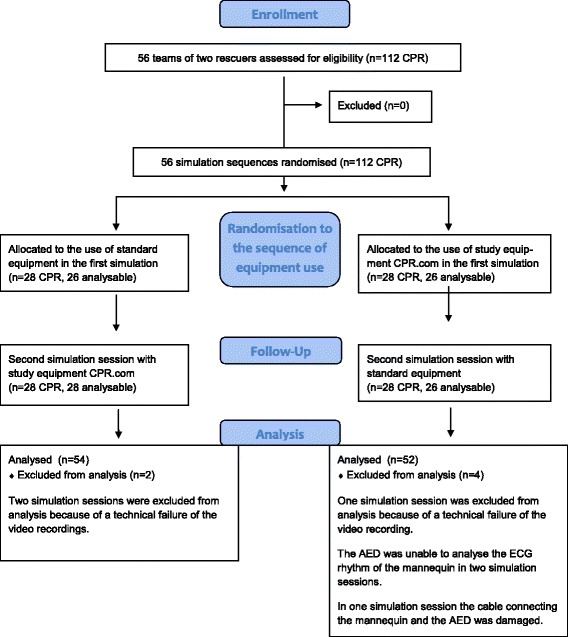
Study flow diagram of allocation to study or standard equipment and data analysis according to CONSORT statement

One hundred twelve participants were included in the study and formed 56 teams of two ALS providers. According to the crossover design of the study, the teams were randomised to 56 simulation sessions with the study equipment and 56 simulation sessions with standard equipment. Of the 112 consecutive simulations that were initially recorded in the study, a complete data set was analysed from 106 simulations (Fig. 2). The characteristics of the participants in the study are presented in Table 1.

**Table 1:** Characteristics of the study participants

Number of participants analysed	106
Duration of each CPR simulation session (min)	8.2 (±0.4)
Female gender	29 (26%)
Age (yrs)	26 (±7.1)
Professional experience as EMS staff member (yrs)	4.0 (±5.8)
Cumulative number of CPR trainings of both team members within the last five years	8.4 (±3.9)
Paramedic	28 (25%)
EMT in vocational training to become paramedic	63 (56%)
EMT not in vocational training to become paramedic	21 (19%)

The data are presented as means (standard deviation) for continuous data and numbers (%) for categorical data

*EMS* emergency medical service; *EMT* emergency medical technician

In this simulation study, the mean hands-off time as the primary outcome did not differ significantly between the simulations with the study equipment CPR.com and those with the standard equipment (Tables 2 and 3).

**Table 2:** Quality of CPR achieved with the study device CPR.com and the standard equipment

	CPR.com device	Standard equipment	Difference between the equipment	*p*-value
Hands-off time (%)	24.5 (23.3 to 25.6)	23.5 (22.3 to 24.6)	1.0 (−0.4 to 2.5)	0.156
Time interval until the first defibrillation (s)	70.3 (64.7 to 75.9)	75.1 (69.4 to 80.8)	−4.8 (−12 to 3)	0.204
Time interval until the second defibrillation (s)	214 (107 to 221)	215 (209 to 222)	−1 (−11 to 8)	0.786
Time interval until the third defibrillation (s)	355 (347 to 364)	352 (343 to 361)	3 (−8 to 14)	0.574
Time interval until intubation of the mannequin with the LT (s)	129 (113 to 146)	121 (104 to 137)	9 (−11 to 28)	0.368
Time interval until the implementation of venous access (s)	305 (290 to 320)	304 (289 to 320)	1 (−21 to 22)	0.946
Time interval until the administration of adrenaline (s)	380 (369 to 392)	403 (391 to 416)	−23 (−37 to −8)	0.003
Time interval until the administration of amiodarone (s)	427 (416 to 438)	444 (432 to 457)	−17 (−31 to −3)	0.016
Number of CC per minute (n)	79 (75 to 82)	82 (78 to 85)	−3 (−5 to −0.4)	0.022
Frequency of CC [min^−1^] (n)	105 (100 to 109)	107 (102 to 111)	−2 (−5 to 0)	0.068
Number of ventilations per minute (n)	6.9 (6.3 to 7.5)	8.7 (8.1 to 9.3)	−1.8 (−2.6 to −1.0)	<0.001
Number of changes of the person doing chest compressions (n)	2.7 (2.5 to 2.9)	2.1 (1.9 to 2.3)	0.6 (0.3 to 0.8)	<0.001

Results are adjusted for the effect of the number of cumulative prior CPR trainings of a rescuer team and presented as means with 95% confidence intervals (95% CI)

Hands-off time: percentage of time without chest compressions during the entire CPR time

*LT* Laryngeal tube; *CC* chest compressions

**Table 3:** Effects of the use of CPR.com device or standard equipment compared with prior CPR training

	Effect of allocation to CPR.com device	Effect of each prior CPR training within the last 5 years
Hands-off time (%)	1.02 (0.7); *p* = 0.156	−0.25 (0.1); *p* = 0.042
Time interval until the administration of adrenaline (s)	−22.85 (7.3); *p* = 0.003	−1.84 (1.3); *p* = 0.157
Time interval until the administration of amiodarone (s)	−16.96 (6.7); *p* = 0.016	−2.99 (1.3); *p* = 0.022
Number of CC per minute [min^−1^] (n)	−2.79 (1.2); *p* = 0.022	−0.02 (0.4); *p* = 0.954
Number of ventilations per minute [min^−1^] (n)	−1.79 (0.4); *p* < 0.001	0.02 (0.1); *p* = 0.774
Number of changes of the person doing chest compressions (n)	0.58 (0.1); *p* < 0.001	0.00 (0.0); *p* = 0.989

Hands-off time: percentage of time without chest compressions during the entire CPR time; *CC* chest compressions

Each row shows the effects determined by an analysis of covariance (ANCOVA) adapted for the cross-over study design. The effect of the allocation to the use of CPR.com study device or standard equipment is presented as marginal means and associated standard errors. The impact of the number of prior CPR trainings is presented as estimates and standard error for the effect of each cumulative prior CPR training of a rescuer team member within the last 5 years

**Fig. 3:**
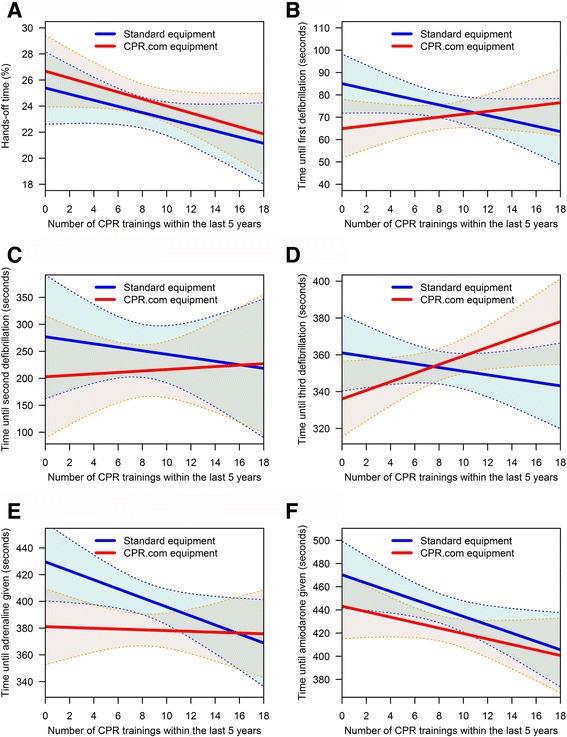
Effect plots illustrating the associations between the allocation to the equipment used (CPR.com device vs. standard equipment) and the cumulative number of prior CPR trainings of both team members within the last 5 years and the hands-off time (**a**), the time until the first defibrillation (**b**), the time until the second defibrillation (**c**), the time until the third defibrillation (**d**), the time interval until the administration of adrenaline (**e**) and the time interval until the administration of amiodarone (**f**). Coloured areas represent the estimator’s confidence bands. Although the plots illustrate the observed different effects of the equipment used and the number of prior CPR trainings of the rescuer teams, the interdependency between the equipment and the number of prior CPR trainings only reached statistical significance at the time of the third defibrillation in the ERC algorithm (**d**) (*p* = 0.014)

The cumulative number of prior CPR trainings attended within the last 5 years by both team members demonstrated a small effect on the hands-off time, i.e., a 0.25% point reduction per prior CPR training of one of the team members (Table 3, Fig. 3). Accordingly, 20 cumulative prior CPR trainings for both team members within the last 5 years (i.e., two trainings per each EMS provider per year) led to a decrease of 5% points in the hands-off time.

Within the secondary endpoints, the study equipment increased the number of changes of the person doing CC and reduced the time interval until the first administration of adrenaline and amiodarone. However, the use of the study equipment resulted in a reduced number of CC and ventilations per minute compared to the standard equipment (Tables 2 and 3).

Furthermore, the CPR.com equipment affected the time that the participants needed to pass through the ERC algorithm until the third defibrillation, but this effect was subject to a significant interaction with the cumulative number of prior CPR trainings (*p* = 0.014, Fig. 3). Figure 3 illustrates the association among the equipment used, the number of prior CPR trainings of the rescuer teams, the hands-off time and the time intervals that the participants needed to pass through the ERC algorithm.

In the simulation sessions with the standard equipment, the time until the first, second and third defibrillations and that until the administration of adrenaline and amiodarone decreased with the number of prior CPR trainings of the participants. In contrast, in the simulation sessions with the study device, the time until the defibrillations increased with the number of prior CPR trainings of the participants. Those teams with only two cumulative CPR trainings in the last five years needed shorter times until defibrillation with the study device than did the teams using the standard equipment (Fig. 3). However, those teams with 18 cumulative CPR trainings in the last five years needed longer times until the defibrillations with the study device compared with the standard equipment (Fig. 3). The analyses showed that teams were faster in the ERC algorithm until the first and third defibrillations with the study device compared to the standard equipment unless they had attended more than approximately 10 cumulative ALS trainings within the last five years per two-person team, which corresponds to one training per participant per year.

**Fig. 4:**
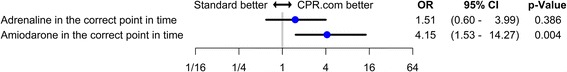
Forest plot of the administration of adrenaline and amiodarone at the correct time according to ERC guidelines as determined with binary logistic regression analyses accounting for cross-over design

Logistic regression revealed that the chance of administering amiodarone at the appropriate time according to the ERC guidelines was approximately four times higher with the use of the study equipment CPR.com compared to the standard equipment (*p* = 0.004). The chance of administering adrenaline at the appropriate time showed an advantage for the CPR.com device as well, but the results did not differ significantly between the study device and the standard equipment (*p* = 0.386) (Fig. 4).

There was no significant effect of the level of vocational education of the participants on the quality of CPR.

A comprehensive dataset with the data analysed in the study is available from the corresponding author on request.

## Discussion

In this simulation study, an interactive prototype guiding defibrillation and ventilation in ALS was used safely by experienced EMS staff after a short introduction to the device.

The participants performed high-quality CPR with both the study and standard equipment compared to other study results. The hands-off times of 24.5 and 23.5%, respectively, were within the same range of the hands-off times reported in other studies (between 16 and 30%) [5, 10, 19–21]. In this study, the new prototype device was not superior to the standard equipment with respect to the hands-off time, whereas the study device slightly decreased the number of CC and ventilations per minute. However, although the differences between the study and standard equipment showed statistically significant effects, these effects are supposed to be of little clinical relevance to the patient’s outcome. The cause for failing to prove superiority might be the lack of familiarity with the equipment and the complexity of the scenario. A previous study by Rittenberger and colleagues showed that EMS focus not enough on BLS in a complex resuscitation scenario [22]. It remains unclear, whether the observed significant differences gain clinical importance in routine use. Some previous studies have found a positive association between the chest compression fraction (CCF) and survival, whereas others found diverging or inconsistent results and concluded that CCF alone is not a predictor of survival of OHCA [1, 21, 23–25]. In the present study, the hands-off time decreased with the number of prior CPR trainings of the participants but did not change with the use of the study device. There is evidence from clinical studies that the greatest chance of survival after OHCA is with CC rates between 100 and 120/min, which the participants achieved with both types of equipment [2, 26].

The increased number of changes of the person doing chest compressions of 0.6 per CPR session might have contributed to longer hands-off times. However, the ERC guidelines recommend changing the person doing chest compressions every 2 min to maintain high-quality CPR, and therefore, the higher number of rescuer’s changeovers might be of clinical relevance [3, 13].

The interaction with the device, which requested performing actions and confirming their completion, may have required time and intention that the EMS staff had available for CC while using the standard equipment. It is unknown whether the results would have been the same if the EMS staff were as familiar with the study device as they were with the standard equipment, which most of them use in daily practice.

In regard to the secondary endpoints, we found a significant interaction between the effect of the CPR.com device and the number of prior CPR trainings in the two-rescuer teams. As shown in Fig. 3, there was an association between the times that the participants needed to work through the ALS algorithm of the ERC, the allocation to the use of the CPR.com device or standard equipment and the cumulative number of prior CPR trainings of both team members within the last 5 years. Participants with fewer prior CPR trainings were faster with the study device than with standard equipment, whereas well-trained participants were possibly slowed down by the study device compared with the standard equipment. These well-trained rescuers needed more time to work through the algorithm with the study device than with the standard equipment. We refer these findings to the fact that by using the study equipment ALS measures were performed only after the CPR.com device prompted them and that the participants had to confirm the completion of an ALS measure on the device’s user interface. Thereby the need to interact with the study device extended the time that the rescuers needed to work through the ALS algorithm. On the other hand, an interactive device guiding the rescuer team through the ALS algorithm is of major help for rescuers who have had little regular training [17].

The time until the administration of adrenaline was shorter with the CPR.com device than with standard equipment, but it was not proven that adrenaline was administered at the correct time more frequently according to the ERC algorithm. This effect can be explained by the study device prompting the participants to prepare adrenaline for iv-injection in advance of the correct time for adrenaline injection. In some cases, this instruction led the participants to administer adrenaline before the CPR.com device indicated the correct time for the adrenaline injection. A better-timed adrenaline injection might be achieved if the device is used regularly. The present study device was designed to guide the rescuer through the ALS algorithm of current guidelines.

However, our results support the recommendations of the 2015 ERC guidelines on the use of prompt devices, which is to use CPR prompt devices only as part of a broader system of CPR quality improvement initiatives rather than as an isolated intervention [3, 13].

The present study has some limitations. First, the CPR quality was evaluated on a mannequin and not in real-life routine patient care with prolonged resuscitation efforts. As in every simulation study, the standardised study setting tries to control for confounders and thereby increases the reliability and validity of the results. On the other hand, the standardised study setting limits transferability of the results into clinical practice. Second, the depth and release of CC and the ventilation’s tidal volumes were not analysed in this simulation study, because according to the manufacturer of the mannequin these data were not validly measured by the software version used in the study when performing simultaneous CC and ventilations as recommended during ALS. A further limitation of the study is the fact that ventilator settings in both, study and standard equipment, were set to standard ventilator settings of critically ill patients. Although the pre-set tidal volume of 500 ml corresponds well to resuscitation guidelines, the ventilation frequency of 12/min used in the study differs from ERC resuscitation guidelines, which recommend 10 breaths/min [13]. Nevertheless, these settings can easily be adjusted in the study device as well as in the standard equipment to better comply with resuscitation guidelines.

Additionally, although the EMS staff members included in this study were professional rescuers working in an urban area with a high frequency of patient care with CPR, they had a mean of only 4 years of professional experience and thereby the participants might be considered inexperienced. Besides, some participants could only report a limited number of CPR trainings within the last 5 years with a mean of 4 years of professional experience in EMS. However, the CPR performance by EMS staff with comparable experience has been evaluated successfully in a similar simulation study and is supposed to be the group profiting the most from such an interactive device [27].

## Conclusion

The study device CPR.com improved the ALS quality of EMS staff with a limited number of prior ALS trainings, but it somehow reduced the ALS quality of providers who were well trained in ALS. Given the limitations of a simulation study, the interactive study device can only be recommended for less trained rescuers and not for rescuers who are training in and performing CPR on a regular basis. EMS crew members may potentially benefit from this assist device, if they have limited experience in CPR, for example during their professional training, or if they have a low frequency of CPR performance, for example in rural areas.

## Abbreviations

AED: Automated external defibrillator
ALS: Advanced life support
ANCOVA: Analyses of covariance
CC: Chest compressions
CI: Confidence interval
CPR: Cardiopulmonary resuscitation
ECG: Electrocardiography
EMS: Emergency medical system
EMT: Emergency medical technician
ERC: European Resuscitation Council
IPPV: Intermittent positive pressure ventilation
LT: Laryngeal tube
OHCA: Out-of-hospital cardiac arrest
PEEP: Positive end expiratory pressure
Pmax: Maximum inspiratory pressure
SD: Standard deviation
SE: Standard error
VF: Ventricular fibrillation
Vt: Tidal volume
